# A Temperature-Ultraviolet-Responsive Fluorescent Anti-Counterfeiting Hydrogel

**DOI:** 10.3390/gels12070634

**Published:** 2026-07-16

**Authors:** Tian Yu, Zhong-Xiang Tang, Meng Jin, Hui Ren, Bin Wu, Yu-Zhuo Fan, Ze-Hui Bai, Fang-Chang Tsai, Xue-Qing Zhan, Ning Ma

**Affiliations:** Hubei Key Laboratory of Polymer Materials, Key Laboratory for the Green Preparation and Application of Functional Materials (Ministry of Education), Hubei Collaborative Innovation Center for Advanced Organic Chemical Materials, School of Materials Science and Engineering, Faculty of Life Sciences, Hubei University, Wuhan 430062, China; 202321113012421@stu.hubu.edu.cn (T.Y.); 202321113012286@stu.hubu.edu.cn (Z.-X.T.); mengjin@stu.hubu.edu.cn (M.J.); 202321113012402@stu.hubu.edu.cn (H.R.); 202321123012712@stu.hubu.edu.cn (B.W.); yuzhuo@stu.hubu.edu.cn (Y.-Z.F.); bzh1015@stu.hubu.edu.cn (Z.-H.B.); m18272163856@163.com (X.-Q.Z.)

**Keywords:** ZIF-8, fluorescein, fluorescence, anti-counterfeiting hydrogel

## Abstract

Information security and anti-counterfeiting are crucial across various industries. To address the limitations of traditional anti-counterfeiting materials, including low responsiveness, easy replication, and poor environmental stability, a fluorescein (Flu) loaded zeolitic imidazolate framework (ZIF-8) photothermal-responsive anti-counterfeiting hydrogel was designed. Flu was first confined within ZIF-8 via a one-pot method and then embedded into a polyacrylamide/lauryl methacrylate (PAM/LMA) network. This hydrogel emits intense green luminescence under 365 nm UV illumination. Its fluorescence can be quenched by Fe^3+^ and recovered upon exposure to PO_4_^3−^, which endows the material with rewritable data storage capacity. Sodium dodecyl sulfate (SDS) and sodium chloride (NaCl) in the hydrogel provide a temperature-dependent reversible transparency transition, allowing multi-level information encryption through the synergistic action of temperature, ions, and UV light. In addition, the hydrogel also features low toxicity, degradability, and an environmentally friendly solvent-free synthesis. This work demonstrates a multi-stimuli responsive strategy that overcomes the limitation of traditional single-responsive anti-counterfeiting materials, offering a promising approach for the design of rewritable and eco-friendly intelligent anti-counterfeiting systems and serving as a reference for the development of multifunctional responsive materials.

## 1. Introduction

With the continuous advancement of counterfeiting and decryption technologies, conventional information storage and anti-counterfeiting systems are facing increasing challenges in terms of security and reliability [1,2,3]. Traditional dry or rigid materials, such as holographic labels, magnetic stripes, and printed codes, typically encode information in static and single-mode format, lacking dynamic tunability and being prone to replication and decoding. Moreover, information storage systems based on a single stimulus responsive (e.g., light-responsive only or temperature-responsive only) are insufficient to establish robust multilevel security barriers, failing to meet the growing demands for advanced information security [4,5]. Thus, smart anti-counterfeiting materials with hierarchical encryption and tunable dynamic response are greatly needed.

Intelligent polymer hydrogels as soft materials with three-dimensional crosslinked network structures have attracted widespread attention due to their structural designability, excellent biocompatibility, and diverse stimulus responsiveness. Distinct from conventional dry and rigid materials, hydrogels can produce physical or chemical color variations upon external stimuli, including light, heat, ions and pH, which grants them distinctive merits in the field of dynamic information encryption [6,7,8,9,10]. Thus, hydrogels combining physical and chemical color-changing effects can be fabricated to increase the anti-counterfeiting threshold. Physical color change modulates the transparency or color of hydrogels through light scattering, refraction and other optical effects [11,12]. Thermoresponsive hydrogels tune their optical properties via the hydrophilic-hydrophobic transition of polymer chains. Their stimulus response requires no complex reagents, with the advantages of simple operation and favorable repeatability. Chemical color change is realized by embedding carbon dots, organic dyes or lanthanide metal ions into the hydrogel system. In recent years, intelligent fluorescent hydrogels constructed by incorporating carbon dots (CDs), organic dyes or lanthanide ions into hydrogel matrices have attracted increasing attention in information security [13,14,15]. Their fluorescence signals can be modulated by external stimuli to enable information writing, storage, and erasure. For instance, Lv et al. introduced CDs into the hydrogel via free radical polymerization to fabricate the fluorescent composite hydrogel, in which information storage was achieved through ion printing for anti-counterfeiting applications [16]. Xiong et al. grafted coumarin derivatives onto chitosan via Schiff-base reactions or amide-acid condensation reactions, yielding a series of chitosan-based fluorescent hydrogels that functioned as fluorescent sensors for the selective recognition, detection and adsorption of Fe^2+^ ions in aqueous solutions [17]. Zhao et al. employed 1,3,5-benzenetricarboxylic acid (H_3_BTC) as the ligand and tuned the Tb^3+^/Eu^3+^ molar ratio to fabricate luminescent hydrogels exhibiting green, red and yellow emissions for anti-counterfeiting [18]. However, aggregation of emissive components within hydrogel networks and their long-term exposure to environmental conditions often lead to fluorescence quenching, which remains a major obstacle to their reliable application in advanced information encryption and anti-counterfeiting systems.

To address the aggregation-induced fluorescence quenching and limited stability of emissive components in fluorescent hydrogels, metal–organic frameworks (MOFs) have attracted increasing attention as an effective structural platform. MOFs are crystalline porous materials formed via coordination-driven self-assembly between metal ions and organic ligands [19,20,21], featuring high porosity and large specific surface area, with well-defined and tunable pore architectures capable of confining and protecting guest nanomaterials [22]. Luminescent MOFs (LMOFs), as an important subclass of MOFs, are a category of materials with excellent luminescent properties [23,24]. Their luminescent characteristics originate from the penetration and loading of guest molecules in the free spaces inside the porous structure of LMOFs [25,26]. Owing to the spatial confinement effect of MOFs, the incorporation of luminescent guests (e.g., carbon dots, organic fluorescent dyes) into their pore systems effectively inhibits the aggregation of guest molecules and minimizes direct environment exposure, thereby reducing unfavorable intermolecular interactions, enhancing fluorescence intensity and stability, and alleviating aggregation-caused quenching (ACQ) effects [27,28,29]. For example, Yang et al. and Zhu et al. embedded CDs into zinc-based MOFs to construct high-performance fluorescent sensors for the detection of metal cations [30,31]. In addition, Xu et al. fabricated a series of single-phase dye@In-MOF phosphors via in situ synthesis and impregnation methods for WLEDs (white-light-emitting diodes) [32].

**Scheme 1:**
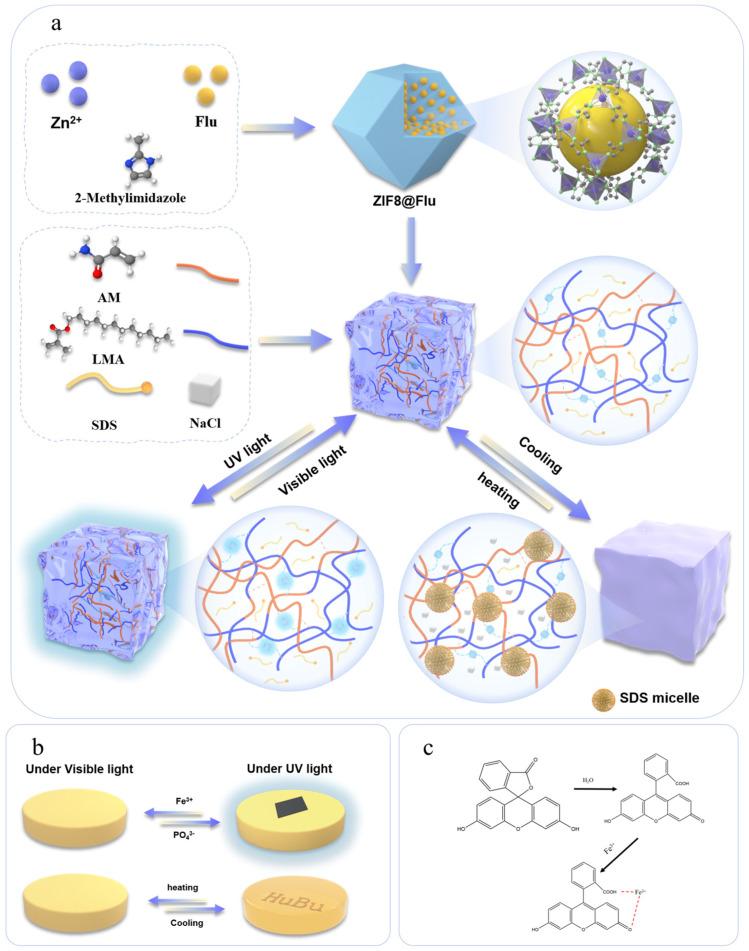
(**a**) Fabrication process diagram of the hydrogel. (**b**) Applications of the hydrogel. (**c**) Fluorescence Quenching Mechanism of Flu [33,36].

Considering the high cost of lanthanide ions and the complexity associated with carbon dots synthesis, fluorescein, a commercially available, low-cost and biocompatible fluorophore, was selected in this study. Fe^3+^ is a well-established fluorescence quencher that readily accepts electrons from electron-rich moieties. After fluorescein dissolves in water, the carbonyl and carboxyl groups on its molecule endow it with electron-rich character, enabling it to donate electrons to Fe^3+^. Accordingly, fluorescein can chelate Fe^3+^ to generate ground-state complexes, which introduce non-radiative decay pathways and thereby reduce or completely quench the fluorescence emission of fluorescein upon interaction with Fe^3+^ [33]. ZIF-8, featuring a high specific surface area and favorable biocompatibility, was employed as a host matrix to improve the stability and photobleaching resistance of fluorescein [34]. Here, a dual-responsive anti-counterfeiting hydrogel with temperature- and fluorescence-sensitive behaviors (PAM/LMA/ZIF-8@Flu) was constructed by incorporating Flu-loaded ZIF-8 and SDS into the PAM/LMA copolymer network (Scheme 1). The hydrogel exhibits a reversible transparency-opaqueness transition in response to temperature variations. Meanwhile, its Flu can be reversibly modulated through coordination with Fe^3+^ and subsequently restored in the presence of PO_4_^3−^, enabling rewritable information storage. By integrating temperature-regulated optical changes, ion-induced fluorescence switching, and UV excitation, a multilevel temperature-ion-UV encryption system was established. Since the global energy crisis, countries around the world have vigorously promoted the philosophy of green development [35]. The PAM/LMA matrix endows the hydrogel with good biocompatibility and potential biodegradability, while the solvent-free fabrication process further enhances its environmental friendliness. Furthermore, the proposed hydrogel system partially overcomes the limitations of traditional anti-counterfeiting materials, providing a promising strategy for the design of high-security and sustainable intelligent anti-counterfeiting materials.

## 2. Results and Discussion

**Figure 1:**
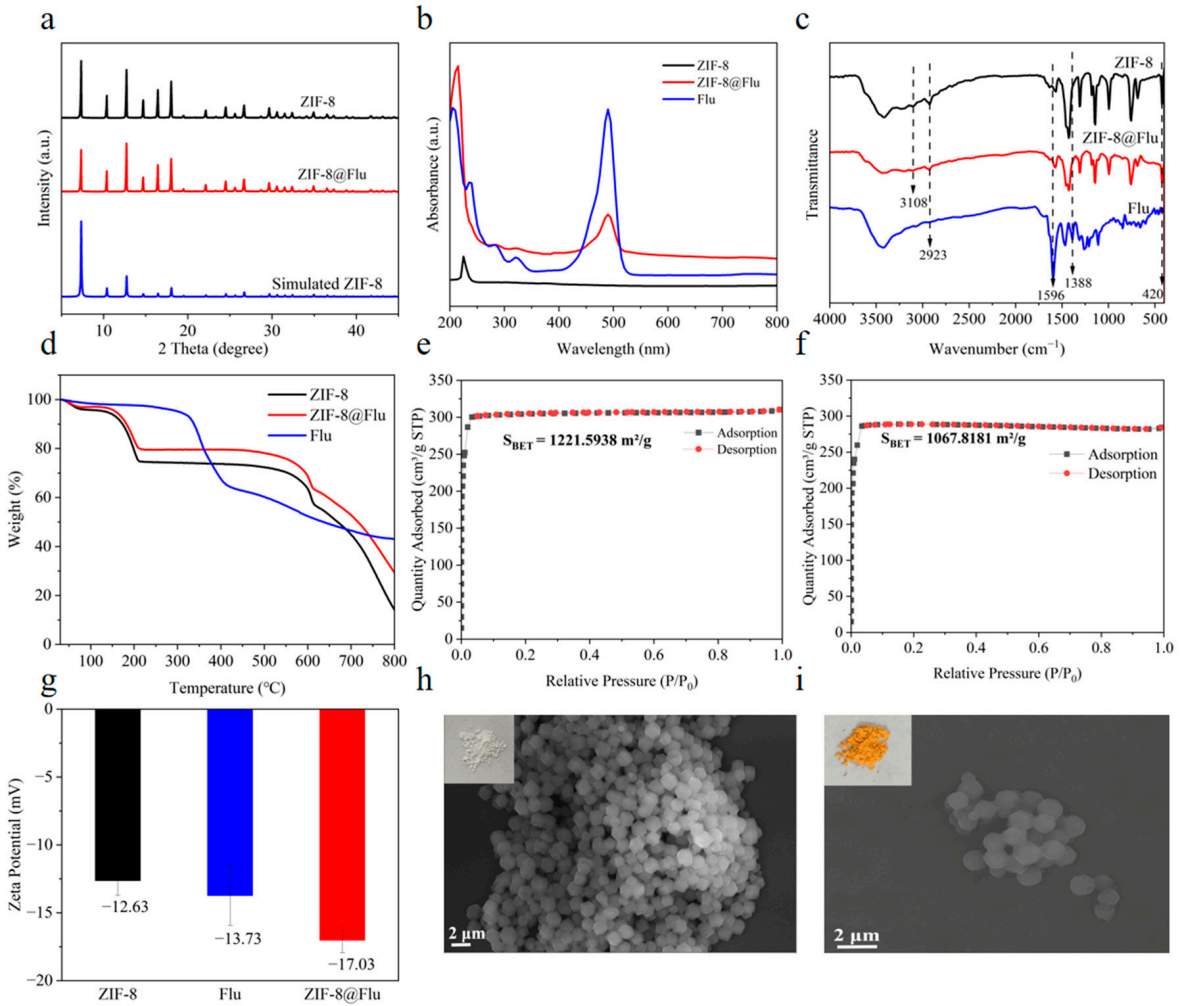
(**a**) XRD spectra of pristine ZIF-8, ZIF-8@Flu composite and simulated ZIF-8 crystal. (**b**) The UV–vis absorption spectra of pristine ZIF-8, pure Flu, and ZIF-8@Flu composite. (**c**) FTIR characteristic spectra of pristine ZIF-8, free Flu and ZIF-8@Flu composite. (**d**) TGA curves of pristine ZIF-8, free Flu and ZIF-8@Flu composite. N_2_ adsorption and desorption isotherms of (**e**) ZIF-8 and (**f**) ZIF-8@Flu; (**g**) Zeta potentials of pristine ZIF-8, free Flu and ZIF-8@Flu composite. SEM images of (**h**) ZIF-8 and (**i**) ZIF-8@Flu.

Structural characterizations were carried out to verify the effective immobilization of Flu within ZIF-8 frameworks. XRD patterns (Figure 1a) reveal that both as-prepared pure ZIF-8 and ZIF-8@Flu composites exhibit nearly identical crystal structures, which match well with the simulated diffraction data of ZIF-8. ZIF-8, Flu and ZIF-8@Flu were separately dispersed in deionized water at a concentration of 0.5 mg/mL. The solutions were diluted multiple times for UV–vis measurements over a scanning wavelength range of 200–800 nm. These results demonstrate that target crystalline products with high purity are obtained, while almost no miscellaneous diffraction peaks can be observed. Figure 1b displays the UV–vis absorption profiles of ZIF-8@Flu, where a wide absorption band centered at 490 nm can be detected, which further evidences the successful embedding of Flu molecules inside ZIF-8. FTIR spectroscopy revealed that ZIF-8@Fluretains a structure similar to ZIF-8 (Figure 1c). The stretching vibration of -OH functional groups generates a broad characteristic peak between 3400 and 3500 cm^−1^. The absorption signal at 2925 cm^−1^ originates from C-H stretching modes of methyl groups [37]. Meanwhile, the signals at 3108 cm^−1^ and 420 cm^−1^ are ascribed to imidazole ring vibrations and Zn-N bond stretching, in sequence [38,39]. The vibrational bands at 1388 cm^−1^ and 1596 cm^−1^ stem from benzene skeleton vibrations and carbonyl (C=O) stretching of Flu molecules, respectively [40,41]. The distinctive band at 420 cm^−1^ verifies the intermolecular interaction between Zn^2+^ cations and Flu. In general, the combination of ZIF-8 and Flu primarily relies on noncovalent forces between imidazole moieties and phenyl groups [42]. Thermogravimetric analysis (TGA) was adopted to assess the thermal stability properties of pure ZIF-8, free Flu and ZIF-8@Flu composite (Figure 1d). Flu began to decompose at 318 °C, whereas ZIF-8 and ZIF-8@Flu showed significant decomposition around 548 °C. The loading of Flu notably increased the initial decomposition temperature of the composite, indicating that the ZIF-8 framework provided thermal protection and delayed Flu decomposition. The thermal weight-loss profiles of ZIF-8@Flu integrate the thermal decomposition features of ZIF-8 organic ligands and the thermal degradation behavior of free Flu, with overall loss intermediate between the two components, verifying the successful loading of Flu and the structural integrity of the composite. As shown in Figure 1e,f, ZIF-8 exhibits persistently superior adsorption performance compared with the ZIF-8@Flu composite. The reduced adsorption capacity of ZIF-8@Flu suggests that the functionalized molecules (Flu) may have penetrated into the pore channels of ZIF-8, thereby diminishing the effective space available for nitrogen adsorption. As illustrated in the Zeta potential diagram (Figure 1g), pristine ZIF-8, Flu and ZIF-8@Flu possess surface charge values of −12.63 mV, −13.73 mV and −17.03 mV separately. These subtle shifts in potential provide solid evidence that Flu molecules are loaded into ZIF-8 cavities. As observed from SEM images (Figure 1h,i), both pristine ZIF-8 and Flu-loaded ZIF-8 exhibit homogeneous 1 μm rhombic dodecahedral structures. No structural damage occurs after the encapsulation of Flu, further corroborating the findings of XRD and FTIR tests.

**Figure 2:**
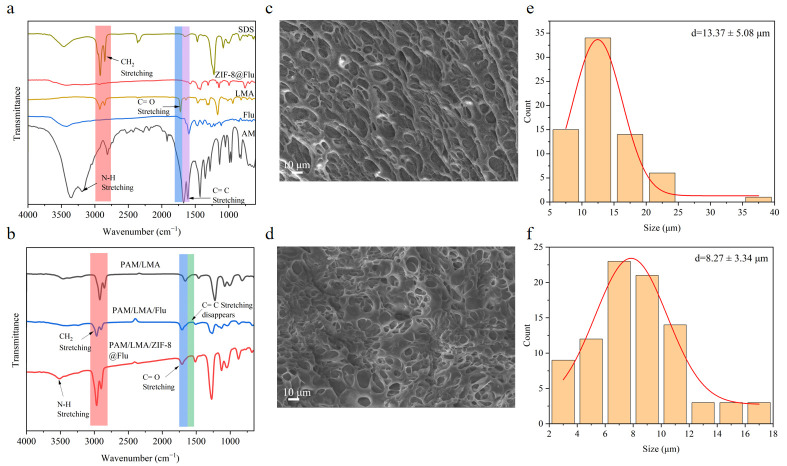
(**a**) FTIR spectra of SDS, ZIF-8@Flu, LMA, Flu and AM. (**b**) FTIR spectra of PAM/LMA, PAM/LMA/Flu and PAM/ZIF-8@Flu hydrogels. SEM images of (**c**) PAM/LMA and (**d**) PAM/ZIF-8@Flu hydrogels. Particle size distribution diagrams of (**e**) PAM/LMA and (**f**) PAM/ZIF-8@Flu hydrogels.

FTIR spectroscopy and SEM observation were employed to probe the chemical constitution and microscopic morphology of the synthesized hydrogels. Figure 2a,b display FTIR spectral curves. The broad absorption band spanning 3200–3400 cm^−1^ originates from N-H stretching vibrations of AM monomers. The signal at 2925 cm^−1^ is associated with C-H stretching modes. The characteristic band near 1650 cm^−1^ derives from C=C stretching belonging to both AM and LMA units, and the peak located at 1722 cm^−1^ corresponds to carbonyl (C=O) stretching vibrations of LMA. Following hydrogel crosslinking, the characteristic N-H stretching vibration of AM was observed at 3500 cm^−1^. The absorption signals at 2962 cm^−1^ and 2896 cm^−1^ originate from the -CH_2_- stretching modes generated by SDS and LMA. Importantly, the vanishing C=C stretching peaks of AM and LMA confirm the complete polymerization reaction, which enables the construction of a stable hydrogel network structure [43]. SEM images of lyophilized hydrogel samples (Figure 2c,d) reveal that all samples exhibit a dense honeycomb-like porous structure. For comparison, the ZIF-8-incorporated hydrogel displays a higher structural density, accompanied by enhanced molecular chain entanglement and cross-linking (Figure 2e,f), indicating that the incorporation of ZIF-8@Flu contributes to the construction of a more robust hydrogel network. Figure S1 displays TGA curves for investigating the thermal resistance of hydrogel materials. It can be observed that adding Flu molecules promotes faster thermal breakdown of the PAM/LMA hydrogel network, whereas encapsulation of Flu within ZIF-8 effectively improves the thermal stability of the hydrogel, owing to the protective confinement effect and network reinforcement.

**Figure 3:**
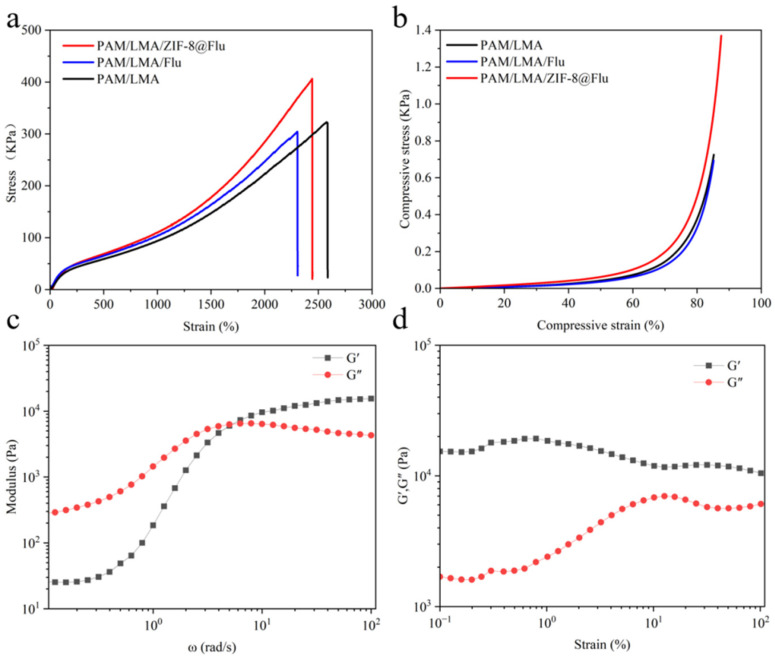
(**a**) Tensile stress–strain curves of the prepared hydrogels. (**b**) Compression stress–strain curves of the prepared hydrogels. (**c**) Dynamic Oscillatory Frequency Sweep and (**d**) Dynamic Oscillatory Strain Sweep of PAM/ZIF-8@Flu hydrogel.

The mechanical properties of PAM/LMA, PAM/LMA/Flu, and PAM/LMA/ZIF-8@Flu hydrogels were evaluated by tensile and compressive tests, as shown in Figure 3a,b. Compared with the pristine PAM/LMA hydrogel, the incorporation of ZIF-8@Flu slightly reduced the fracture strain but markedly increased the tensile stress to 400 kPa, indicating effective reinforcement of the hydrogel network. The enhancement is attributed to ZIF-8@Flu acting as rigid fillers and multi-point physical crosslinking sites, which strengthen polymer chain interactions and improve stress transfer, while partially restricting chain mobility. In compression, the ZIF-8@Flu-containing hydrogel sustained 1.3 kPa at 85% strain, reflecting superior compressive resistance due to the homogeneous dispersion of ZIF-8@Flu that suppresses localized stress concentration. Rheological analysis of the PAM/LMA/ZIF-8@Flu hydrogel further elucidated the reinforcement mechanism. As shown in Figure 3c, at low angular frequencies, the loss modulus (G″) was comparable to or exceeded the storage modulus (G′), indicating a predominantly viscous response. With increasing frequency (ω > 5 rad/s), G′ surpassed G″, showing a transition to elastic-dominated behavior. The distinct viscoelastic transition point (ω ≈ 5 rad/s) reflects a typical elastic-like characteristic of the hydrogel network. Across 0.1–100 Hz, G′ remains consistently higher than G″, with both moduli rising synchronously (Figure 3d), demonstrating that the hydrogel network maintains structural stability under broad dynamic conditions. These results confirm that the incorporation of ZIF-8@Flu enhances both static mechanical strength and dynamic elastic stability.

**Figure 4:**
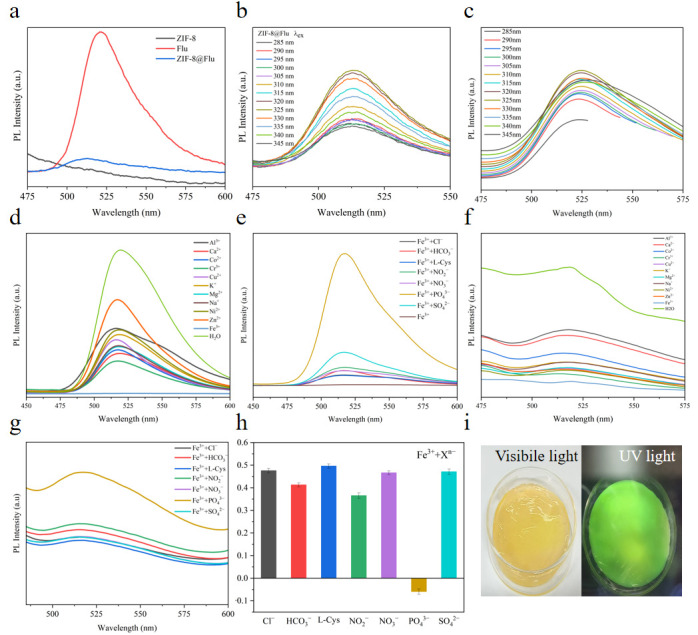
(**a**) Fluorescent spectral curves recorded for ZIF-8, Flu and Flu-loaded ZIF-8 (*λ_ex_* = 325 nm). (**b**) Luminescence intensity of ZIF-8@Flu and (**c**) PAM/LMA/ZIF-8@Flu hydrogel recorded under varied excitation wavelengths. (**d**) Emission profiles of ZIF-8@Flu incubated in diverse metal ion solutions and (**e**) Fe^3+^ solutions containing various anions (*λ_ex_* = 325 nm). (**f**) Fluorescent spectra of PAM/LMA/ZIF-8@Flu hydrogel treated with different metal cations and (**g**) Fe^3+^ mixtures with distinct anions (*λ_ex_* = 325 nm). (**h**) Histogram illustrating the fluorescence intensity ratio (I_F0_ − I_F_)/I_F0_ of PAM/LMA/ZIF-8@Flu hydrogel exposed to various metal cations (All subsequent fluorescence measurements were conducted in triplicate, with error bars representing standard deviation). (**i**) State diagram under visible light and UV light of PAM/LMA/ZIF-8@Flu hydrogel.

Fluorescence spectrophotometry was utilized to explore the luminescence behaviors of pristine ZIF-8, free Flu and ZIF-8@Flu composite, as displayed in Figure 4. ZIF-8, Flu and ZIF-8@Flu were separately dissolved in deionized water at a concentration of 0.2 mg/mL, and fluorescence measurements were performed on the resulting solutions. Under excitation at 325 nm, ZIF-8 exhibited negligible fluorescence emission, while Flu showed a strong emission peak at 520 nm (Figure 4a). In contrast, ZIF-8@Flu displayed an intense green emission peak at 513 nm, indicating that the fluorescence originated from Flu rather than ZIF-8. In line with the above results, ZIF-8@Flu water solution presents an orange color in a visible environment and produces green fluorescence when exposed to 365 nm ultraviolet light. The excitation wavelength-dependent luminescence performance of ZIF-8@Flu was also measured within 285–345 nm (measurements were carried out on aqueous ZIF-8@Flu solutions with a concentration of 0.2 mg/mL), as illustrated in Figure 4b. The strongest excitation signal was recorded at 325 nm, corresponding to a maximum emission peak at 513 nm. Accordingly, 325 nm was chosen as the optimal excitation wavelength for all follow-up tests. Afterwards, we assessed the impacts of diverse metal ions on the luminescence intensity of ZIF-8@Flu (Figure 4d). Upon exposure to solutions containing Ca^2+^, Ni^2+^, K^+^, Al^3+^, Mg^2+^, Cu^2+^, Na^+^, Co^2+^, Fe^3+^, Cr^3+^, and Zn^2+^ (0.001 M), respectively, a pronounced fluorescence quenching was observed exclusively in the presence of Fe^3+^, demonstrating high selectivity toward Fe^3+^. A similar fluorescence response was observed for the PAM/LMA/ZIF-8@Flu hydrogel. When excited at 325 nm, the hydrogel exhibited a maximum emission at 515 nm (Figure 4c), consistent with the behavior of ZIF-8@Flu. After immersion in various metal ion solutions (0.01 M), only Fe^3+^ induced significant fluorescence quenching of the hydrogel (Figure 4f). Subsequently, the Fe^3+^-quenched hydrogels were immersed in solutions containing Cl^−^, HCO_3_^−^, L-Cysteine (L-Cys), NO_2_^−^, NO_3_^−^, PO_4_^3−^, and SO_4_^2−^ (0.1 M), respectively (Figure 4g,h). Notably, fluorescence recovery was observed only in the presence of PO_4_^3−^, indicating a reversible and selective fluorescence response toward the Fe^3+^/PO_4_^3−^. In addition, the agar diffusion assay was performed to assess the antibacterial property of the prepared hydrogels, with Gram-positive Staphylococcus aureus serving as the model bacterial strain (Figure S2). Distinct inhibition zones were observed around all hydrogel samples, indicating effective antibacterial performance. The antibacterial capability significantly enhances the practical applicability of the hydrogels and broadens their potential application scope.

**Figure 5:**
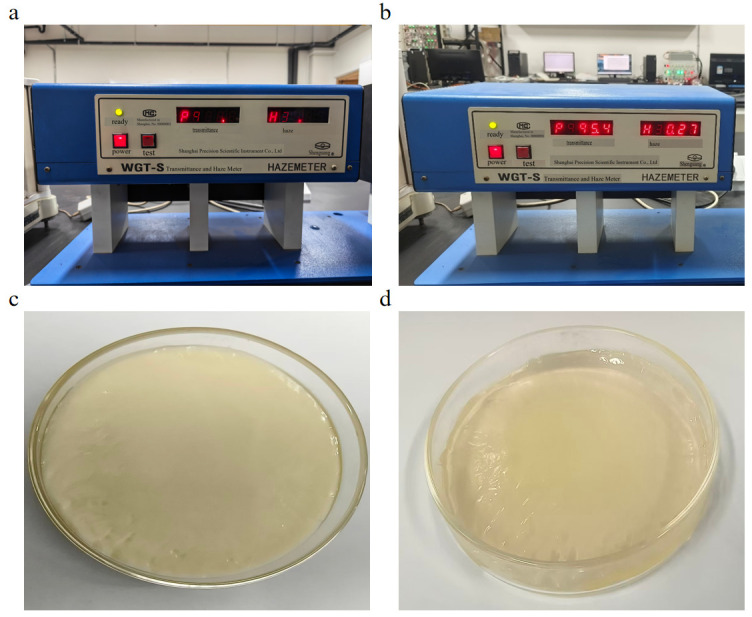
PAM/LMA/ZIF-8@Flu hydrogel (**a**) transmittance/haze diagram at 8 °C; (**b**) transmittance/haze diagram at 37 °C; (**c**) digital image under visible light at 8 °C; (**d**) digital image under visible light at 37 °C.

The temperature sensitivity of the hydrogel was evaluated using a transmittance/haze meter (Figure 5). At 8 °C, the instrument displayed no transmittance or haze value, and this result was identical to that obtained when the test port was covered with an opaque cap (no light transmission), indicating that the hydrogel was opaque at low temperatures. In contrast, at 37 °C, the PAM/LMA/ZIF-8@Flu hydrogel (thickness: 3 mm) exhibited a transmittance of 95.4% and a haze of 0.27, allowing the bottom contour of the hydrogel to be clearly observed. These results demonstrate that the hydrogel becomes highly transparent with low haze upon heating. Transmittance and haze testing results therefore verify that the PAM/LMA/ZIF-8@Flu hydrogel exhibits an opaque state at low temperatures and turns transparent to visible light upon heating. This thermally responsive behavior originates from the crystallization of sodium chloride (NaCl) under low-temperature conditions. The formed inorganic crystals promote the micellization of SDS, thereby endowing the hydrogel with opacity. Upon heating, the NaCl crystals dissolve, leading to micelle dissociation and recovery of transparency [44]. This reversible switching between opaque and transparent states, combined with the UV fluorescence response of ZIF-8@Flu, provides a core platform for constructing temperature-UV dual-responsive hydrogels, enabling multi-level anti-counterfeiting functionality.

**Figure 6:**
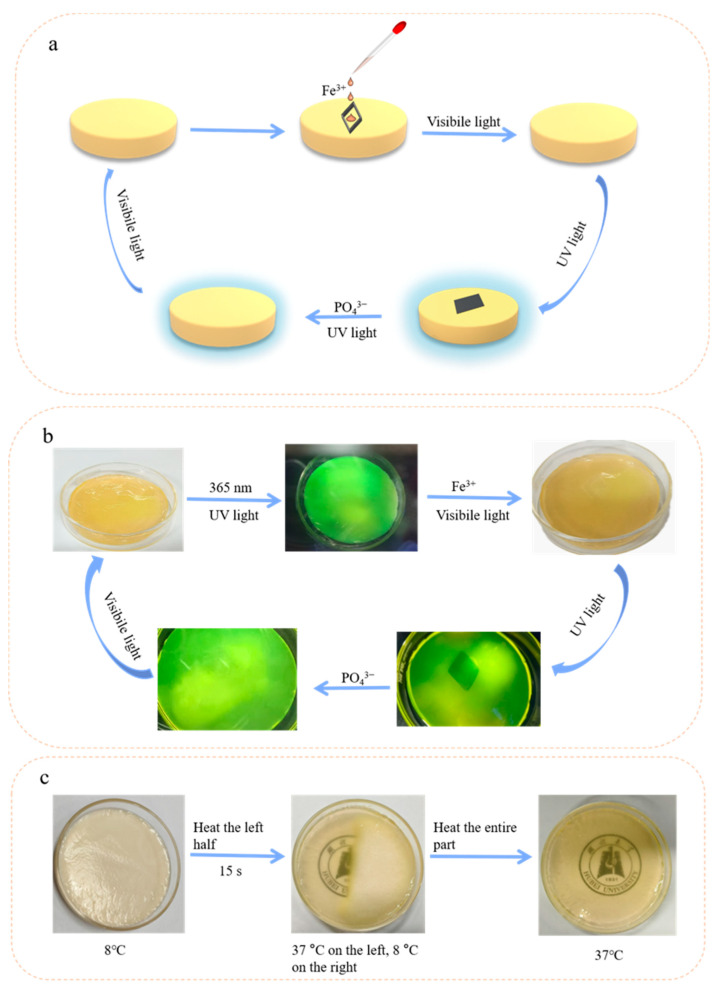
Information recording and anti-counterfeiting applications of PAM/LMA/ZIF-8@Flu hydrogel relying on (**a**) process flow diagram, (**b**) fluorescence-switching behavior, and (**c**) temperature-responsive optical transparency.

Benefiting from Fe^3+^-triggered fluorescence quenching and reversible luminescence restoration mediated by PO_4_^3−^, the PAM/LMA/ZIF-8@Flu hydrogel system achieves information storage functionality (Figure 6a). In this process, an ion-brushing strategy was employed, where the Fe^3+^ solution served as “ink” and the hydrogel acted as “writing substrate”. As shown in Figure 6b, when a Fe^3+^ impregnated template was placed in contact with the hydrogel, localized diffusion of Fe^3+^ ions induced fluorescence quenching, making the encoded patterns invisible under visible light yet distinctly observable upon 365 nm ultraviolet excitation. Subsequent treatment with a PO_4_^3−^ solution restored the fluorescence through competitive coordination with Fe^3+^, effectively erasing the original information and enabling rewritable data storage. This demonstrates that PAM/LMA/ZIF-8@Flu hydrogel possesses both rewritable and self-erasing information storage. The fluorescence writing-erasing switching relies on the reversible competitive coordination between Fe^3+^ and phosphate ions within the hydrogel. In theory, this chemical interaction can be repeated many times without fundamental damage to the fluorescent substrate. During multiple cycles, slight attenuation of fluorescence intensity occurs due to the accumulation of trace ions confined in the gel network. Meanwhile, owing to the presence of SDS, the hydrogel exhibits a reversible temperature-dependent transition between transparency and opaque states (Figure 6c), introducing an additional level of visual concealment.

## 3. Conclusions

In conclusion, a temperature-fluorescence dual-stimuli responsive anti-counterfeiting hydrogel, PAM/LMA/ZIF-8@Flu, was successfully developed. By confining Flu within the pores of ZIF-8 and incorporating it into a PAM/LMA copolymer network, a three-dimensional hydrogel with stable fluorescence and favorable mechanical properties was obtained. Encapsulating Flu within the ZIF-8 framework exerts no influence on its crystalline structure while greatly boosting luminescence performance. The fluorescence of the hydrogel could be reversibly quenched by Fe^3+^ and subsequently restored in the presence of PO_4_^3−^. enabling rewritable information storage. In addition, SDS endowed the hydrogel with a temperature-dependent reversible transparency transition: the hydrogel is opaque at low temperatures, concealing information embedded within the bulk, and becomes transparent upon heating. This is because inorganic salt crystallization induces SDS to form micelles at low temperatures, rendering the hydrogel opaque; at elevated temperatures, micelles dissociate and crystals dissolve, restoring the hydrogel’s transparency. By integrating ion-responsive fluorescence switching, thermal optical modulation, and UV excitation, this system enables multi-level encryption and decryption, offering a promising strategy for green, rewritable and high-security intelligent anti-counterfeiting materials.

## 4. Materials and Methods

### 4.1. Materials

Acrylamide (AM), lauryl methacrylate (LMA), fluorescein (Flu), sodium dodecyl sulfate (SDS), zinc acetate dihydrate (Zn(CH_3_COO)_2_·2H_2_O, analytical grade), copper(II) chloride dihydrate (CuCl_2_·2H_2_O, analytical grade), chromium(III) chloride (CrCl_3_, analytical grade), cobalt(II) nitrate hexahydrate (Co(NO_3_)_2_·6H_2_O, analytical grade), 2-methylimidazole (C_4_H_6_N_2_, 98%) and N, N′-methylenebisacrylamide (MBA) were all commercially sourced from Aladdin Reagent Co., Ltd., Shanghai, China. Ammonium persulfate (APS), anhydrous calcium chloride (CaCl_2_, analytical grade), nickel(II) chloride hexahydrate (NiCl_2_·6H_2_O, analytical grade), potassium chloride (KCl, analytical grade), aluminum(III) chloride hexahydrate (AlCl_3_·6H_2_O, analytical grade), sodium chloride (NaCl, analytical grade), and iron(III) chloride hexahydrate (FeCl_3_·6H_2_O, analytical grade) were provided by Sinopharm Chemical Reagent Co., Ltd., Beijing, China. Magnesium chloride dihydrate (MgCl_2_, analytical grade) was obtained from Shanghai Yien Chemical Technology Co., Ltd., Shanghai, China. Zinc chloride (ZnCl_2_, analytical grade) and L-Cysteine (L-Cys, C_3_H_7_NO_2_S, 99%) were purchased from Shanghai Macklin Biochemical Technology Co., Ltd., Shanghai, China. Laboratory-produced deionized water was adopted as the experimental solvent throughout the whole experiment. All chemical reagents in this work were utilized directly after delivery with no additional purification treatment.

### 4.2. Preparation of ZIF-8

2-Methylimidazole (2.442 g) and zinc acetate dihydrate (Zn(CH_3_COO)_2_·2H_2_O, 1.2 g) were individually dissolved in 20 mL of deionized water to prepare solution A and solution B, respectively. Afterwards, solution A was dropwise added into solution B gradually, and the resultant mixed system was fully homogenized before static incubation for 10 h. After the reaction was completed, the resultant product was washed and dried sequentially, and the final ZIF-8 sample was harvested for subsequent use.

### 4.3. Preparation of ZIF-8@Flu

2-Methylimidazole (2.442 g) and Flu (0.332 g) were co-dissolved in 20 mL of deionized water to formulate homogeneous solution A. Meanwhile, zinc acetate dihydrate (Zn(CH_3_COO)_2_·2H_2_O, 1.2 g) was dispersed in another 20 mL of deionized water to yield solution B. Thereafter, solution A was slowly incorporated into solution B, and the blended mixture was fully stirred evenly before standing for 10 h at ambient conditions. The obtained precipitate was then subjected to washing and drying treatments, and the final ZIF-8@Flu composite was collected after processing.

### 4.4. Preparation of PAM/LMA Hydrogel

SDS (2.1 g) and NaCl (1.4 g) were dispersed in 30 mL of deionized water, followed by continuous stirring at 50 °C to achieve a transparent and homogeneous solution. After that, 0.5 mL of LMA was introduced into the above system, and the mixture was stirred continuously for 2 h. Afterwards, AM (3 g), APS (0.11 g), and MBA (0.006 g) were added in sequence, with continuous blending for 10 min to obtain a uniform reaction precursor solution. The resultant mixture was finally kept statically at 60 °C for 10 min to complete gelation, thereby forming the PAM/LMA hydrogel.

### 4.5. Preparation of PAM/LMA/Flu Hydrogel

SDS (2.1 g) and NaCl (1.4 g) were dispersed in 30 mL of deionized water, followed by continuous stirring at 50 °C to achieve a transparent and homogeneous solution. After that, 0.5 mL of LMA was introduced into the above system, and the mixture was stirred continuously for 2 h. Afterwards, AM (3 g), Flu (0.001 g), APS (0.11 g), and MBA (0.006 g) were added in sequence, with continuous blending for 10 min to obtain a uniform reaction precursor solution. The resultant mixture was finally kept statically at 60 °C for 10 min to complete gelation, thereby forming the PAM/LMA/Flu hydrogel.

### 4.6. Preparation of PAM/LMA/ZIF-8@Flu Hydrogel

SDS (2.1 g) and NaCl (1.4 g) were dispersed in 30 mL of deionized water, followed by continuous stirring at 50 °C to achieve a transparent and homogeneous solution. After that, 0.5 mL of LMA was introduced into the above system, and the mixture was stirred continuously for 2 h. Afterwards, AM (3 g), ZIF-8@Flu (0.02 g), APS (0.11 g), and MBA (0.006 g) were added in sequence, with continuous blending for 10 min to obtain a uniform reaction precursor solution. The resultant mixture was finally kept statically at 60 °C for 10 min to complete gelation, thereby forming the PAM/LMA/ZIF-8@Flu hydrogel.

### 4.7. Characterizations

X-ray diffraction (XRD, D8 Advance, Bruker AXS GmbH, Karlsruhe, Germany) was utilized to characterize the crystalline structures of pure ZIF-8 and ZIF-8@Flu composites. Fourier transform infrared spectroscopy (FTIR, Nicolet iS50, Thermo Fisher, Waltham, Massachusetts, USA) was employed to investigate the chemical components and molecular structural features of the prepared hydrogels. The surface morphology and microscopic architectures of the samples were visualized via a field emission scanning electron microscope (SEM, Sigma 500, Carl Zeiss AG, Oberkochen, Germany). Thermogravimetric analysis (TGA, TA-SDTQ600, TA Instruments, New Castle, DE, USA) was performed to assess the thermal stability and thermal decomposition behaviors of the materials. A TU-1810DSPC spectrophotometer (Persee, Beijing, China) was used to collect ultraviolet-visible (UV-vis) absorption spectra. The Zeta potential of the samples was tested using a Zetasizer Nano ZS90 instrument (Malvern Panalytical Ltd., Malvern, UK). At ambient temperature, an F-320 fluorescence spectrophotometer (Tianjin Gangdong Sci. & Tech. Co., Ltd., Tianjin, China) was adopted to record the fluorescence emission signals, while the fluorescence lifetime parameters were measured by a FluoTime 300 fluorescence spectrometer (PicoQuant GmbH, Berlin, Germany).

### 4.8. Tensile Testing of Hydrogels

Mechanical tensile tests were carried out on an Instron 68TM-10 high and low-temperature universal testing machine (Instron Corporation, Norwood, MA, USA). Rectangular hydrogel specimens with dimensions of 0.8 cm in width, 0.3 cm in thickness and 5 cm in length were measured at room temperature. The tests were conducted with a 50 N load cell at a stretching rate of 200 mm/min.

### 4.9. Rheological Property of Hydrogels

A DH-2 advanced rotational rheometer (TA Instruments, New Castle, DE, USA) equipped with 25 mm parallel plates was applied for rheological characterization. The testing gap was set to 1 mm, and all measurements were implemented at 25 °C. Dynamic oscillatory frequency sweep tests were conducted under a fixed strain of 1%, with the angular frequency ranging from 0.1 to 100 rad/s.

### 4.10. Fluorescence Property of PAM/LMA/ZIF-8@Flu in Different Solutions

The selective fluorescence response of PAM/LMA/ZIF-8@Flu hydrogels toward diverse metal ions and phosphate radicals (PO_4_^3−^) was explored through systematic immersion experiments. Cylindrical hydrogel samples with a diameter of 14 mm and a height of 6 mm were placed in 24-well plates. Each well was filled with 1 mL of solution, including deionized water serving as the blank group and 0.01 M aqueous solutions containing different metal ions (Ca^2+^, Ni^2+^, K^+^, A^3+^, Cr^3+^, Mg^2+^, Cu^2+^, Na^+^, Co^2+^, Fe^3+^ and Zn^2+^). After 10 min of immersion, the fluorescence selectivity of the hydrogel was assessed. For fluorescence recovery measurements, hydrogel samples pre-treated with Fe^3+^ solution were further soaked in 1 mL of 0.1 M PO_4_^3−^ solution for another 10 min. All fluorescence emission spectra of the above-treated hydrogels were acquired under a fixed excitation wavelength of 325 nm.

### 4.11. Information Storage of PAM/LMA/ZIF-8@Flu

Non-stick paper was tailored into designated shapes using scissors to fabricate customized printing templates, which were then attached to the hydrogel surface. The hollow patterned areas of the prefabricated templates were uniformly coated with 0.01 M Fe^3+^ solution, enabling the spontaneous diffusion of Fe^3+^ ions into the hydrogel matrix to quench its fluorescence performance. After peeling off the non-stick paper, a distinct patterned structure was successfully constructed on the hydrogel surface. The patterned hydrogel was subsequently immersed in PO_4_^3−^ solution for fluorescence recovery treatment. Ultimately, a camera was utilized to record and analyze the optical variations in the hydrogel under visible light and 365 nm ultraviolet (UV) light irradiation.

## Data Availability

The data that support the findings of this study are available from the corresponding author upon reasonable request.

## Supplementary Materials

The following supporting information can be downloaded at: https://www.mdpi.com/article/10.3390/gels12070634/s1, Figure S1: TGA curves of PAM/LMA, PAM/LMA/Flu and PAM/LMA/ZIF-8@Flu; Figure S2: Antibacterial activity diagram of PAM/LMA, PAM/LMA/Flu and PAM/LMA/ZIF-8@Flu.

## References

[1] Babazadeh-Mamaqani M., Razzaghi D., Roghani-Mamaqani H., Babaie A., Rezaei M., Hoogenboom R., Salami-Kalajahi M. (2024). Photo-responsive electrospun polymer nanofibers: Mechanisms, properties, and applications. Prog. Mater. Sci..

[2] Shen Y., Le X., Wu Y., Chen T. (2024). Stimulus-responsive polymer materials toward multi-mode and multi-level information anti-counterfeiting: Recent advances and future challenges. Chem. Soc. Rev..

[3] Zhou H., Han J., Cuan J., Zhou Y. (2022). Responsive luminescent MOF materials for advanced anticounterfeiting. Chem. Eng. J..

[4] An X., Song S., Li W., Jiang Z., Fan X., Ma P., Hou H., Zhang Y. (2025). Movable-Type Printing and Chameleon-Inspired Photothermal Bimodal Flexible Polymer Arrays for Spatiotemporally Programmable Multilevel Encryption. Adv. Funct. Mater..

[5] Le X., Shang H., Wu S., Zhang J., Liu M., Zheng Y., Chen T. (2021). Heterogeneous Fluorescent Organohydrogel Enables Dynamic Anti-Counterfeiting. Adv. Funct. Mater..

[6] Amirthalingam S., Rajendran A.K., Moon Y.G., Hwang N.S. (2023). Stimuli-responsive dynamic hydrogels: Design, properties and tissue engineering applications. Mater. Horiz..

[7] Sun Y., Le X., Zhou S., Chen T. (2022). Recent Progress in Smart Polymeric Gel-Based Information Storage for Anti-Counterfeiting. Adv. Mater..

[8] Wei S., Li Z., Lu W., Liu H., Zhang J., Chen T., Tang B.Z. (2020). Multicolor Fluorescent Polymeric Hydrogels. Angew. Chem. Int. Ed..

[9] Ding X., Yang B., Si X., Ni L., Fang C., Hou Z. (2026). Robust Polyurethane Hydrogels Based on Dynamic Disulfide Bonds and Pendant Tertiary Amines with Room-Temperature Self-Healing and pH Responsiveness. Gels.

[10] Wang S., Wang G., Liu X., Bi J., Xiao W., Wang D., Liu M., Gao C., Xu Z., Wang Z. (2026). Temperature/pH Dual-Responsive Hydrogels: Research Progress in Preparation Methods, Structural Design Strategies and Biomedical Applications. Gels.

[11] Jiang X., Yan P., Wu C., Ke J., Hou W., Huang J., Lian Z., Lü T., Bai L. (2026). Wrinkled Photonic Elastomers with Dynamic Structural Color Patterns for Multilevel Optical Anti-Counterfeiting. Gels.

[12] Qu Z., Wang L., Fang S., Qin D., Zhou J., Yang G., Duan H. (2019). Fluorescein-immobilized optical hydrogels: Synthesis and its application for detection of Hg^2+^. Microchem. J..

[13] Ain N.u., Rehman Z., Ibrahim T.K., Kebaili I., Boukhris I., Al-Buriahi M.S. (2025). Organic dyes-based photothermal ablation of cancer cells: A review. Dye. Pigment..

[14] Ru Y., Waterhouse G.I.N., Lu S. (2022). Aggregation in carbon dots. Aggregate.

[15] Sun C., Gradzielski M. (2022). Advances in fluorescence sensing enabled by lanthanide-doped upconversion nanophosphors. Adv. Colloid Interface Sci..

[16] Lv H., Wang S., Wang Z., Meng W., Han X., Pu J. (2022). Fluorescent cellulose-based hydrogel with carboxymethyl cellulose and carbon quantum dots for information storage and fluorescent anti-counterfeiting. Cellulose.

[17] Xiong S., Duan L., Cheng X. (2020). A novel coumarin-chitosan fluorescent hydrogel for the selective identification of Fe^2+^ in aqueous systems. Polym. Chem..

[18] Zhao S., Gao M., Li J. (2021). Lanthanides-based luminescent hydrogels applied as luminescent inks for anti-counterfeiting. J. Lumin..

[19] Zhu Q.-L., Xu Q. (2014). Metal–organic framework composites. Chem. Soc. Rev..

[20] Yue N., Umer S., Subramaniam R., Teoh W.Y., Yang C. (2025). Emerging Metal–Polymer Frameworks: Structure, Applications, and Perspectives. Adv. Funct. Mater..

[21] Ma X., Xiong Y., Liu Y., Han J., Duan G., Chen Y., He S., Mei C., Jiang S., Zhang K. (2022). When MOFs meet wood: From opportunities toward applications. Chem.

[22] Shu Y., Ye Q., Dai T., Xu Q., Hu X. (2021). Encapsulation of Luminescent Guests to Construct Luminescent Metal–Organic Frameworks for Chemical Sensing. ACS Sens..

[23] Yang J., Ni W., Ruan B., Tsai L.-C., Ma N., Shi D., Jiang T., Tsai F.-C. (2021). Review—Design and Synthesis of Fluorescence Sensing Metal-Organic Frameworks. ECS J. Solid State Sci. Technol..

[24] Zhang K.-D., Tsai F.-C., Ma N., Xia Y., Liu H.-L., Zhan X.-Q., Yu X.-Y., Zeng X.-Z., Jiang T., Shi D. (2017). Adsorption Behavior of High Stable Zr-Based MOFs for the Removal of Acid Organic Dye from Water. Materials.

[25] Gutiérrez M., Zhang Y., Tan J.-C. (2022). Confinement of Luminescent Guests in Metal–Organic Frameworks: Understanding Pathways from Synthesis and Multimodal Characterization to Potential Applications of LG@MOF Systems. Chem. Rev..

[26] Ruan B., Yang J., Zhang Y.-J., Ma N., Shi D., Jiang T., Tsai F.-C. (2020). UiO-66 derivate as a fluorescent probe for Fe^3+^ detection. Talanta.

[27] Zhang Y., Zhao Y., Zhou A., Qu Q., Zhang X., Song B., Liu K., Xiong R., Huang C. (2021). “Turn-on” ratiometric fluorescent probe: Naked-eye detection of acidic pH and citric acid (CA) by using fluorescence spectrum and its application in real food samples and zebrafish. Spectrochim. Acta Part A Mol. Biomol. Spectrosc..

[28] Zhao K., Liu F., Sun H., Xia P., Qu J., Lu C., Zong S., Zhang R., Xu S., Wang C. (2023). A Novel Ion Species- and Ion Concentration-Dependent Anti-Counterfeiting Based on Ratiometric Fluorescence Sensing of CDs@MOF-Nanofibrous Films. Small.

[29] Kaur H., Sundriyal S., Pachauri V., Ingebrandt S., Kim K.-H., Sharma A.L., Deep A. (2019). Luminescent metal-organic frameworks and their composites: Potential future materials for organic light emitting displays. Coord. Chem. Rev..

[30] Yang J., Ruan B., Ye Q., Tsai L.-C., Ma N., Jiang T., Tsai F.-C. (2022). Carbon dots-embedded zinc-based metal-organic framework as a dual-emitting platform for metal cation detection. Microporous Mesoporous Mater..

[31] Zhu C., Yang J., Ni W., Zeng W., Xu J., Zhang K., Zhan X.-Q., Ma N., Tsai F.-C. (2023). Carbon dots-embedded zinc-based metal-organic framework as an efficient fluorescent sensor for the detection of ferric and phosphate ions. J. Environ. Chem. Eng..

[32] Xu D.-D., Dong W.-W., Li M.-K., Han H.-M., Zhao J., Li D.-S., Zhang Q. (2022). Encapsulating Organic Dyes in Metal–Organic Frameworks for Color-Tunable and High-Efficiency White-Light-Emitting Properties. Inorg. Chem..

[33] Khalid R., Javid T., Pervaiz A., Assiri M.A., Khan Z.A., Sania, Shahzad S.A. (2025). AIEE-driven highly sensitive fluorescent probe for Fe^3+^ sensing in aqueous and solid phases: Application in interference-free biological media. RSC Adv..

[34] Zeng W., Jiang Q., Ruan C., Ni W., Zhu C., Zeng X., Shi X., You R., Ma N., Tsai F.-C. (2025). A rewritable and shape memory hydrogel doped with fluorescein-functionalized ZIF-8 for information storage and fluorescent anti-counterfeiting. Talanta.

[35] Ergün M.E., Ergunb H. (2023). Investigating the Feasibility of Guar Gum-Based Foams for Insulation Applications Using Regression Analysis. Dyna.

[36] Chen S., Nakamura H., Tamura Z. (1979). Supplemental studies on relationship between structure and spectrum of fluorescein. Chem. Pharm. Bull..

[37] Lu T., Liang H., Cao W., Deng Y., Qu Q., Ma W., Xiong R., Huang C. (2022). Blow-spun nanofibrous composite Self-cleaning membrane for enhanced purification of oily wastewater. J. Colloid Interface Sci..

[38] Yang C., Du C., Yuan F., Yu P., Wang B., Su C., Zou R., Wang J., Yan X., Sun C. (2024). CRISPR/Cas12a-derived ratiometric fluorescence sensor for high-sensitive Pb^2+^ detection based on CDs@ZIF-8 and DNAzyme. Biosens. Bioelectron..

[39] Zhang X., Li Z., Zhang T., Chen J., Ji W., Wei Y. (2021). Fabrication of an efficient ZIF-8 alginate composite hydrogel material and its application to enhanced copper(ii) adsorption from aqueous solutions. New J. Chem..

[40] Li J., Dai Y., Cui J., Abrha H., Kang N., Liu X. (2023). Dye-encapsulated Zr-based MOFs composites as a sensitive platform for ratiometric luminescent sensing of antibiotics in water. Talanta.

[41] Xiong T., Zhang Y., Donà L., Gutiérrez M., Möslein A.F., Babal A.S., Amin N., Civalleri B., Tan J.-C. (2021). Tunable Fluorescein-Encapsulated Zeolitic Imidazolate Framework-8 Nanoparticles for Solid-State Lighting. ACS Appl. Nano Mater..

[42] Xu N., Tang Z., Jiang Y.-P., Fang J., Zhang L., Lai X., Sun Q.-J., Fan J.-M., Tang X.-G., Liu Q.-X. (2023). Highly Sensitive Ratiometric Fluorescent Flexible Sensor Based on the RhB@ZIF-8@PVDF Mixed-Matrix Membrane for Broad-Spectrum Antibiotic Detection. ACS Appl. Mater. Interfaces.

[43] Wang R., Ma Y., Chen P., Sun L., Liu Y., Gao C. (2023). A double network conductive gel with robust mechanical properties based on polymerizable deep eutectic solvent. Colloids Surf. A Physicochem. Eng. Asp..

[44] Xu G., Xia H., Chen P., She W., Zhang H., Ma J., Ruan Q., Zhang W., Sun Z. (2021). Thermochromic Hydrogels with Dynamic Solar Modulation and Regulatable Critical Response Temperature for Energy-Saving Smart Windows. Adv. Funct. Mater..

