# Supplementary material for: A Temperature-Ultraviolet-Responsive Fluorescent Anti-Counterfeiting Hydrogel
Source: Gels. 2026 Jul 16;12(7):634. doi: 10.3390/gels12070634 (PMC13408535; doi:10.3390/gels12070634)
Supplement: Supplementary file 1 [file gels-12-00634-s001.zip › gels-4384486-supplementary.pdf]

Supporting Information

Figure S1. TGA curves of PAM/LMA, PAM/LMA/Flu and PAM/LMA/ZIF-8@Flu

Figure S2. Antibacterial activity diagram of PAM/LMA, PAM/LMA/Flu and PAM/LMA/ZIF-8@Flu

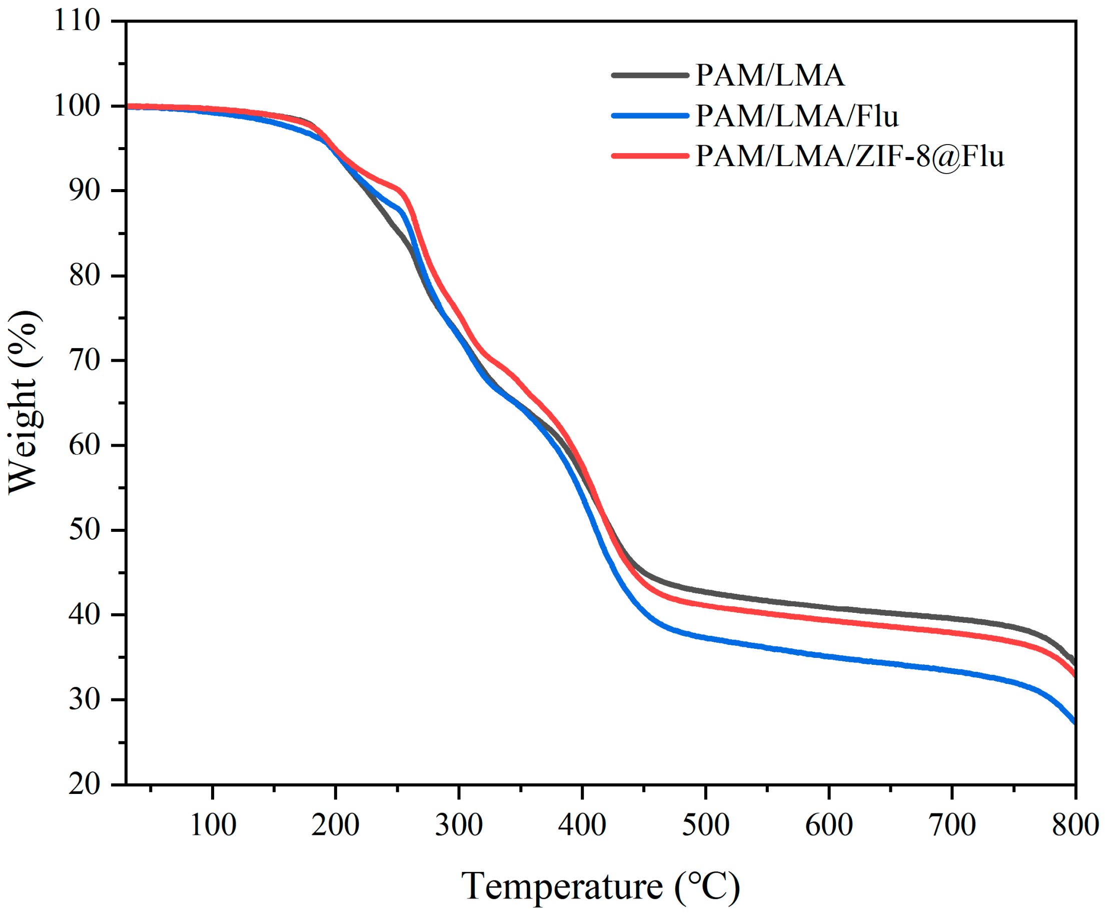

Figure S1. TGA curves of PAM/LMA, PAM/LMA/Flu and PAM/LMA/ZIF-8@Flu

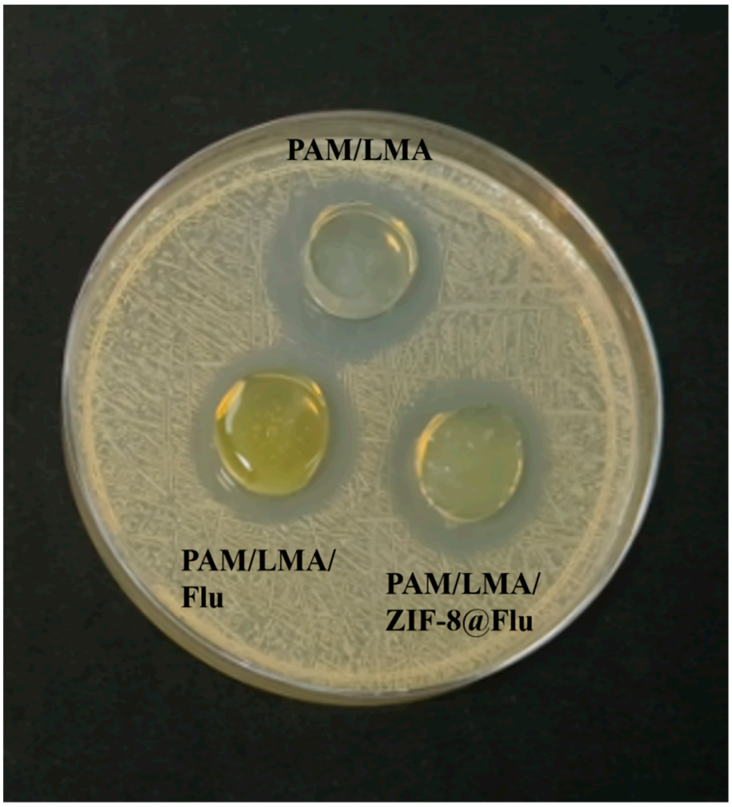

Figure S2. Antibacterial activity diagram of PAM/LMA,PAM/LMA/Flu and PAM/LMA/ZIF-8@Flu
